# wFReDoW: A Cloud-Based Web Environment to Handle Molecular Docking Simulations of a Fully Flexible Receptor Model

**DOI:** 10.1155/2013/469363

**Published:** 2013-04-11

**Authors:** Renata De Paris, Fábio A. Frantz, Osmar Norberto de Souza, Duncan D. A. Ruiz

**Affiliations:** ^1^Laboratório de Bioinformática, Modelagem e Simulação de Biossistemas (LABIO), Faculdade de Informática (FACIN), Pontifícia Universidade Católica do Rio Grande do Sul (PUCRS), Avenida Ipiranga 6681, Prédio 32, Sala 608, 90619-900 Porto Alegre, RS, Brazil; ^2^Grupo de Pesquisa em Inteligência de Negócio (GPIN), Faculdade de Informática (FACIN), Pontifícia Universidade Católica do Rio Grande do Sul (PUCRS), Avenida Ipiranga 6681, Prédio 32, Sala 628, 90619-900 Porto Alegre, RS, Brazil

## Abstract

Molecular docking simulations of fully flexible protein receptor (FFR) models are coming of age. In our studies, an FFR model is represented by a series of different conformations derived from a molecular dynamic simulation trajectory of the receptor. For each conformation in the FFR model, a docking simulation is executed and analyzed. An important challenge is to perform virtual screening of millions of ligands using an FFR model in a sequential mode since it can become computationally very demanding. In this paper, we propose a cloud-based web environment, called web Flexible Receptor Docking Workflow (wFReDoW), which reduces the CPU time in the molecular docking simulations of FFR models to small molecules. It is based on the new workflow data pattern called self-adaptive multiple instances (P-SaMIs) and on a middleware built on Amazon EC2 instances. P-SaMI reduces the number of molecular docking simulations while the middleware speeds up the docking experiments using a High Performance Computing (HPC) environment on the cloud. The experimental results show a reduction in the total elapsed time of docking experiments and the quality of the new reduced receptor models produced by discarding the nonpromising conformations from an FFR model ruled by the P-SaMI data pattern.

## 1. Introduction

Large-scale scientific experiments have an ever-increasing demand for high performance computing (HPC) resources. This typical scenario is found in bioinformatics, which needs to perform computer modeling and simulations on data varying from DNA sequence to protein structure to protein-ligand interactions [1]. The data flood, generated by these bioinformatics experiments, implies that technological breakthroughs are paramount to process an interactive sequence of tasks, software, or services in a timely fashion.

Rational drug design (RDD) [2] constitutes one of the earliest medical applications of bioinformatics [1]. RDD aims to transform biologically active compounds into suitable drugs [3]. *In silico* molecular docking simulation is one of the main steps of RDD. It is used to deal with compound discovery, typically by computationally virtual screening a large database of organic molecules for putative ligands that fit into a binding site [4] of the target molecule or receptor (usually a protein). The best ligand orientation and conformation inside the binding pocket is computed in terms of the free energy of bind (FEB) by software, for instance the AutoDock4.2 [5]. 

In order to mimic the natural, *in vitro* and *in vivo*, behavior of ligands and receptors, their plasticity or flexibility should be treated in an explicit manner [6]: our receptor is a protein that is an inherently flexible system. However, the majority of molecular docking methods treat the ligands as flexible and the receptors as rigid bodies [7]. In this study we model the explicit flexibility of a receptor by using an ensemble of conformations or snapshots derived from its molecular dynamics (MD) simulations [8] (reviewed by [9]). The resulting model receptor is called a fully-flexible receptor (FFR) model. Thus, for each conformation in the FFR model, a docking simulation is executed and analyzed [7].

Organizing and handling the execution and analysis of molecular docking simulations of FFR models and flexible ligands are not trivial tasks. The dimension of the FFR model can become a limiting factor because instead of performing docking simulations in a single, rigid receptor conformation, we must carry out this task for all conformations that make up the FFR model [6]. These conformations can vary in number from thousands to millions. Therefore, the high computing costs involved in using FFR models to perform practical virtual screening of thousands or millions of ligands may make it unfeasible. For this reason, we have been developing methods to simplify or reduce the FFR model dimensionality [6, 9, 10]. We named this simpler representation of an FFR model a reduced fully flexible receptor (RFFR) model. An RFFR model is achieved by eliminating redundancy in the FFR model through clustering its set of conformations, thus generating subgroups, which should contain the most promising conformations [6].

To address these key issues, we propose a cloud-based web environment, called web Flexible Receptor Docking Workflow (wFReDoW), to fast handle the molecular docking simulations of FFR models. To the best of our knowledge, it is the first docking web environment that reduces both the dimensionality of FFR models and the overall docking execution time using an HPC environment on the cloud. The wFReDoW architecture contains two main layers: Server Controller and (flexible receptor middleware) FReMI. Server Controller is a web server that prepares docking input files and reduces the size of the FFR model by means of the self-adaptive multiple instances (P-SaMIs) data pattern [9]. FReMI handles molecular docking simulations of FFR models integrated with an HPC environment on Amazon EC2 resources [11].

There are a number of approaches that predict ligand-receptor interactions on HPC environments using AutoDock4.2 [5]. Most of them use the number of ligands to distribute the tasks among the processors. For instance, DOVIS 2.0 [12] uses a dedicated HPC Linux cluster to execute virtual screening where ligands are uniformly distributed on each CPU. VSDocker 2.0 [13] and Mola [14] are other examples of such systems. Whilst VSDocker 2.0 works on multiprocessor computing clusters and multiprocessor workstations operated by a Windows HPC Server, Mola uses AutoDock4.2 and AutoDock Vina to execute the virtual screening of small molecules on nondedicated compute clusters. Autodock4.lga.MPI [15] and mpAD4 [16] use another approach to enhance the performance. As well as the docking parallel execution, Autodock4.lga.MPI and mpAD4 reduce the quantity of network I/O traffic during the loading of grid maps at the beginning of each docking simulation. Another approach is the AutoDockCloud [17]. This is a high-throughput screening of parallel docking tasks that uses the open source Hadoop framework implementing the MapReduce paradigm for distributed computing on a cloud platform using AutoDock4.2 [5]. Although every one of these environments reduces the overall elapsed time of the molecular docking simulations, they only perform docking experiments with rigid receptors. Conversely, wFReDoW applies new computational techniques [6, 10, 11, 18] to reduce the CPU time in the molecular docking simulations of FFR models using public databases of small molecules, such as ZINC [19].

In this work we present the wFReDoW architecture and its execution. From the wFReDoW executions we expect to find better ways to reduce the total elapsed time in the molecular docking simulations of FFR models. We assess the gains in performance and the quality of the results produced by wFReDoW using a small FFR model clustered by data mining techniques, a ligand from ZINC database [19], different P-SaMI parameters [10], and an HPC environment built on Amazon EC2 [18]. Thus, from the best results obtained, we expect that future molecular docking experiments, with different ligands and new FFR models, will use only the conformations that are significantly more promising [6] in a minimum length of time.

## 2. Methods

### 2.1. The Docking Experiments with an FFR Model

**Figure 1:**
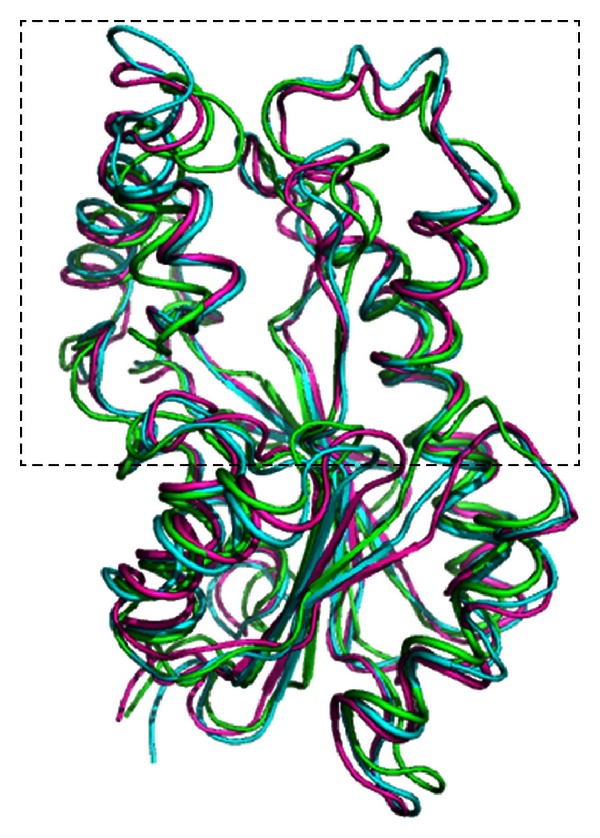
Flexibility of the InhA enzyme receptor from *Mycobacterium tuberculosis* [PDB ID: 1P45A]. Superposition of different InhA conformations, represented as ribbons, along an MD simulation. The initial conformation of the simulation is the experimental crystal structure and is colored in green. Two other conformations or snapshots were taken from the MD simulation at 1,000 ps (blue) and 3,000 ps (magenta). The outlined rectangle highlights the most flexible regions of this receptor.

To perform molecular docking simulations we need a receptor model, a ligand, and docking software. We used as receptor the enzyme 2-*trans*-enoyl-ACP (CoA) reductase (EC 1.3.1.9) known as InhA from *Mycobacterium tuberculosis* [20]. The FFR model of InhA was obtained from a 3,100 ps (1 picosecond = 10^−12^ second) MD simulation described in [21], thus making an FFR model with 3,100 conformations or snapshots. In this study, for each snapshot in the FFR model, a docking simulation is executed and analyzed.  Figure 1 illustrates the receptor flexibility.

**Figure 2:**
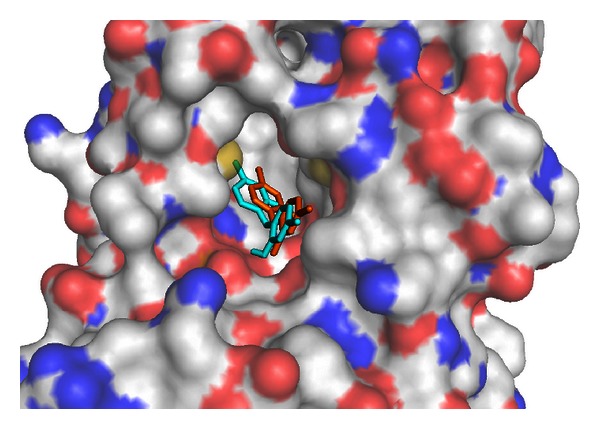
Molecular docking simulation. Molecular surface representation of the binding pocket of the InhA enzyme receptor in the crystal structure [PDB ID: 1P45A] colored by atom type (carbon and hydrogen: light grey; nitrogen: blue; oxygen: red; sulphur: yellow). The TCL ligand (TCL400 from PDB ID: 1P45A) is represented by stick models. The crystallographic reference for the TCL ligand is colored orange. The TCL ligand generated by molecular docking simulation is colored cyan.

The ligand triclosan (TCL400 from PDB ID: 1P45A) [20] was docked to the FFR model. We chose TCL from the referred crystal structure because it is one of the simplest inhibitors cocrystallized with the InhA enzyme.  Figure 2 illustrates the reference position of the TCL400 ligand into its binding site (PDB ID: 1P45A) and the position of the TCL ligand after an FFR InhA-TCL molecular docking simulation.

For docking simulations, we used the AutoDock Tools (ADT) and AutoDock4.2 software packages [5]. Input coordinate files for ligand and the FFR model of InhA were prepared with ADT as follows. (1) Receptor preparation. A PDBQT file for each snapshot from the FFR model was generated employing Kollman partial atomic charges for each atom type. (2) Flexible ligand preparation. The TCL ligand was initially positioned in the region close to its protein binding pocket and allowed two rotatable bonds. (3) Reference ligand preparation. This is the ideal position and orientation of the ligand that is expected from docking simulations. A TCL reference ligand was also prepared using the coordinates of the experimental structure (PDB ID: 1P45A). It is called the reference ligand position. (4) Grid preparation. For each snapshot a grid parameter file (GPF) was produced with box dimensions of 100 Å × 60 Å × 60 Å. The other parameters maintained the default values. (5) Docking parameters. Twenty-five Lamarckian genetic algorithm (LGA) independent runs were executed for each docking simulation. The LGA search method and parameters were: a population size of 150 individuals, a maximum of 250,000 energy evaluations and 27,000 generations. The other docking parameters were kept at default values.

### 2.2. Reducing the Fully Flexible Receptor Model

The snapshots of the FFR model used in this study are derived from an MD simulation trajectory of the receptor. Even though this approach is considered the best to mimic the natural behavior of ligands and receptors [9], its dimension or size may become a limiting factor. Moreover, the high computing cost involved could also make the practical virtual screening of such receptor models unfeasible. For these reasons, new methods have been developed to assist in the simplification or reduction of an FFR model to an RFFR model. The primary rationale of this approach is to eliminate redundancy in the FFR model through clustering of its constituent conformations [6]. This is followed by the generation of subgroups with the most promising conformations via the P-SaMI data pattern [10].

#### 2.2.1. Clusters of Snapshots from an FFR Model

The clusters of snapshots used in this study were generated using clustering algorithms with different similarity functions developed by [6, 7]. Basically, in this approach, our FFR model was used to find patterns that define clusters of snapshots with similar features. In this sense, if a snapshot is associated with a docking with significantly negative FEB, for a unique ligand, it is possible that this snapshot will interact favorably with structurally similar ligands [6]. As a consequence, the clusters of snapshots, which were related to different classes of FEB values, are postprocessed using the P-SaMI data pattern to select the receptor conformations and, thus, to reduce the complexity of the FFR model.

#### 2.2.2. P-SaMI Data Pattern for Scientific Workflow

**Figure 3:**
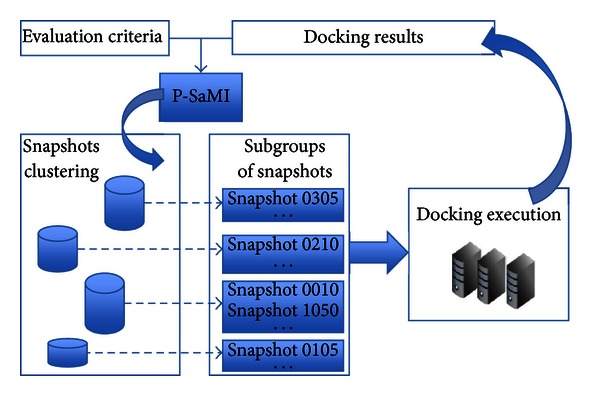
Model of P-SaMI data pattern execution. Clustered snapshots are divided into subgroups using the P-SaMI data pattern. Molecular docking simulations are executed on these subgroups. P-SaMI analyses the docking results, based on some evaluation criteria, to select promising conformations from subgroups of snapshots.

P-SaMI is the acronym for pattern-self-adaptive multiple instances—a data pattern for scientific workflows developed by [10]. The purpose of this approach is to define a data pattern which is able to dynamically perform the selection of the most promising conformations from clusters of similar snapshots. As shown in Figure 3, the P-SaMI first step is to capture a clustering of snapshots from [6]. Next, P-SaMI divides each cluster into subgroups of snapshots to progressively execute *autogrid4* and *autodock4* for each conformation that makes up the FFR model using an HPC environment. The results (*docking results*) are the best FEB value for each docked snapshot. From these results, P-SaMI uses previous FEB results (*evaluation criteria*) to determine the status and priority of the subgroups of snapshots. Status denotes whether a subgroup of snapshots is active (A), finalized (F), discarded (D), or with changed priority (P). Priority indicates how promising the snapshots are belonging to that subgroup, on a scale of 1 to 3 (1 being the most promising). Thus, if the docking results of a subgroup present an acceptable value of FEB then that subgroup is credited with a high priority. Conversely, the subgroup has its priority reduced or its status changed to “D” and is discarded, unless all the snapshots of that subgroup have already been processed (status “F”). 

The reason for using P-SaMI in this work is to make full use of its data pattern to eliminate the exhaustive execution of docking simulations of an FFR model without affecting its quality [6, 10] from clusters of snapshots produced by [6, 7] as input files. In this sense, we make use of a web server environment, herein called server controller, to perform the P-SaMI data pattern and a middleware (FReMI) to handle promising snapshots and send them to an HPC environment on the cloud to execute the molecular docking simulations.

### 2.3. HPC on Amazon EC2 Instances

Cloud computing is a new promising trend for delivering information technology services as computing utilities [22]. Commercial cloud services can play an attractive role in scientific discovery because they provide computer power on demand over the internet, instead of several commodity computers connected by a fast network. Our virtual HPC environment on Amazon EC2 was built using the GCC 4.6.2 and MPICH2 based on a master-slave paradigm [23]. It contains 5 High-CPU extra large (*c1.xlarge*) EC2 Amazon instances, each equipped with 8 cores with 2.5 EC2 computer units, 7 GB of RAM, and 1,690 GB of local instance storage. A rating of one EC2 computer units is a unit of CPU capacity which corresponds to 1.0–1.2 GHZ 2007 Opteron or 2007 Xeon processor. 

**Figure 4:**
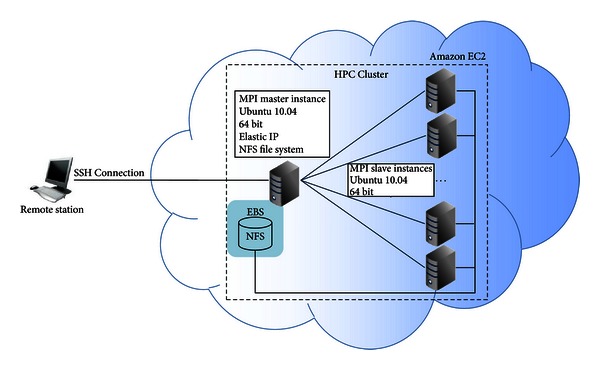
MPI cluster environment created to execute FReMI on Amazon Ec2. The remote station represents the machine outside Amazon EC2 used to connect the MPI master instance by an SSH connection. The MPI master instance is the machine that manages the MPI slaves during the FReMI execution. It also holds the FReMI source code and the I/O files stored on the Amazon Elastic Block Store (EBS). All instances may access EBS through NFS.

Figure 4 shows the cluster pool created on Amazon EC2's instances where the same files directory is shared by network file system (NFS) among the instances to store all input and output files used during run time of FReMI. In this pool, all data are stored on the Elastic Block Store (EBS) of the master machine and all the instances have permission to read and write in this shared directory, even if a slave instance terminates. However, if the master instance terminates, all data are lost because the master instance EBS volume terminates at the same time. Thus, the S3cmd source code (S3cmd is an open source project available under GNU Public License v2 and free for commercial and private use. It is a command line tool for uploading, retrieving, and managing data in Amazon's S3. S3cmd is available at http://s3tools.org/s3cmd) and package is used to replicate the most important information from Amazon EC2 to Amazon S3 bucket (bucket is the space to store data on Amazon S3. Each bucket is identified with a unique bucket name).

## 3. Results 

The results are aimed at showing the wFReDoW architecture and validating its execution using clusters of snapshots of a specific FFR model against a single ligand. From these results we try to evidence that the proposed cloud-based web environment can be more effective than other methods used to automate molecular docking simulations with flexible receptors, such as [24]. In this sense we divided our results into three parts. Firstly, we present the wFReDoW conceptual architecture to get a better understanding about its operation. Next, a set of experiments is examined to discover the best FReMI performance on Amazon EC2 Cloud. Finally, the new RFFR models are presented by means of the wFReDoW execution.

### 3.1. wFReDoW Conceptual Architecture

**Figure 5:**
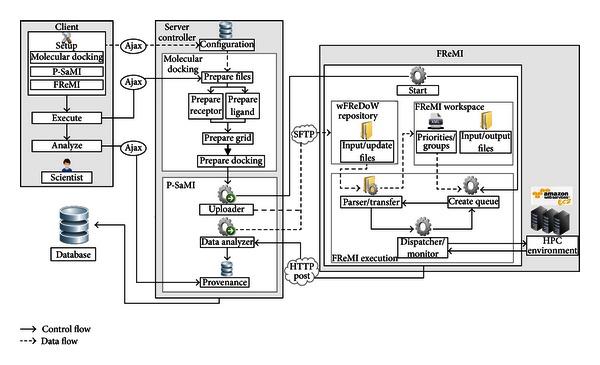
wFReDoW conceptual architecture and its interactions. The two left boxes show the tasks to be executed by a user on the web server, which sends and receives messages to and from FReMI on Amazon EC2. The HPC environment represents the MPI cluster on Amazon EC2.

This section presents the wFReDoW conceptual architecture (Figure 5) which was developed to speed up the molecular docking simulations for clusters of the FFR model's conformations. wFReDoW contains two main layers: Server Controller and FReMI. Server Controller is a web workflow based on P-SaMI data pattern that prepares Autodock input files and selects promising snapshots through docked snapshots. FReMI is a middleware based on the many-task computing (MTC) [25] paradigm that handles high-throughput docking simulations using an HPC environment built on Amazon EC2 instances. In our study, MTC is used to address the problem of executing multiple parallel tasks in multiple processors. Figure 5 details the wFReDoW conceptual architecture with its layers and interactions. The wFReDoW components are distributed in three layers: Client, Server Controller and FReMI.

#### 3.1.1. Client Layer

The Client layer is a web interface used by the scientist to configure the environment. It initializes the wFReDoW execution and analyzes information about the molecular docking simulations. Client is made up of three main components: (i) *Setup* component sets up the whole environment before starting the execution; (ii) *Execute* starts the wFReDoW execution and; (iii) *Analyze* shows the provenance of each docking experiment. The communication between Client and Server Controller is done by means of Ajax (http://api.jquery.com/category/ajax/).

#### 3.1.2. Server Controller

Server Controller is a web workflow environment that aids in the reduction of the execution time of molecular docking simulations of FFR models by means of P-SaMI data pattern. It was built using the web framework FLASK 0.8 (http://flask.pocoo.org/) and the Python 2.6.6 libraries. The Server Controller central role is to select promising subgroups of snapshots from an FFR model based on the P-SaMI data pattern [10]. It contains three components: *Configuration*, *Molecular Docking*, and *P-SaMI*. The *Configuration* component only stores data sent from *Setup* (Client layer).

The *Molecular Docking* component manages the P-SaMI input files and performs the predocking steps required for AutoDock4.2 [5]. Firstly, the *Prepare Files* activity reads the clustering of snapshots generated by [6] and stores them in the *Database*. Next, the *Prepare Receptor* and *Prepare Ligand* activities generate the PDBQT files used as input files to *autogrid4* and *autodock4*. Finally, the *Prepare Grid* and *Prepare Docking* activities create the input files according to the *autogrid4* and *autodock4* parameters, respectively. 

After all files have been prepared by the *Molecular Docking* component, the *P-SaMI* component is invoked. This identifies the most promising conformations using the P-SaMI data pattern [10] from different clusters of snapshots of an FFR model identified by [6]. The *P-SaMI* component contains three activities: *Uploader*, *Data Analyzer*, and *Provenance*. 

**Figure 6:**
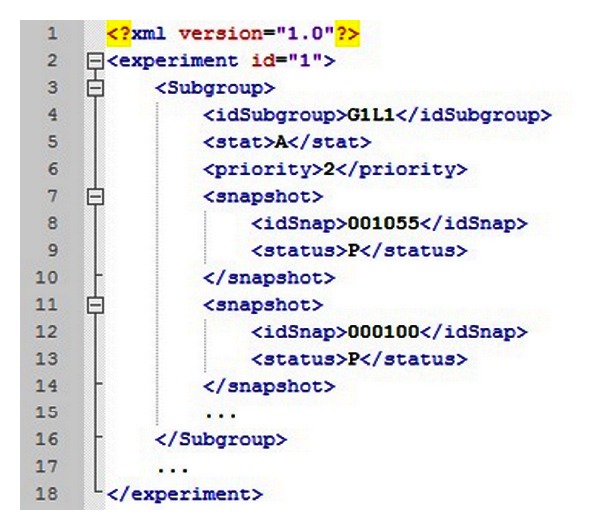
Fragment of the wFReDoW control file. The file places the subgroups of snapshots generated by data mining techniques and its parameters according to P-SaMI.

*Uploader* starts the FReMI execution and generates subgroups from snapshot clustering [6]. These subgroups are stored in an XML file structure, called wFReDoW control file (Figure 6). The wFReDoW control file is sent to the *Parser*/*Transfer* component (within FReMI) before starting the wFReDoW execution. It contains three root tags described as: *experiment*, *subgroup*, and *snapshot*. The experiment identification (id) is a unique number created for each new docking experiment with an FFR model and one ligand. The *subgroup* tag specifies the information of the subgroups. The *stat* and *priority* tags indicate how promising the snapshots belonging to that subgroup are, according to the rules of the P-SaMI data pattern. The *snapshot* tag contains information about the snapshots and is used by FReMI to control the docked snapshots. 

**Figure 7:**
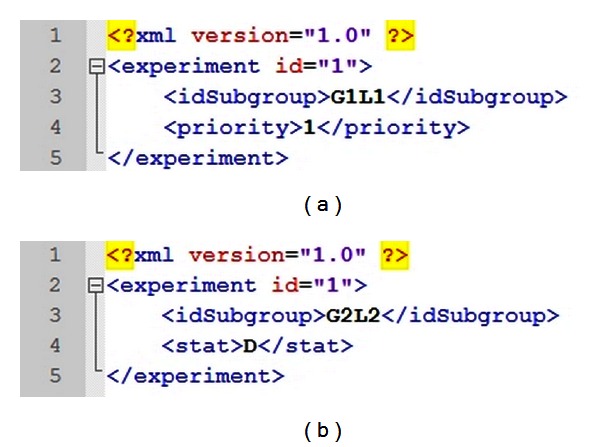
Examples of wFReDoW update files. (a) An XML file where the priority from G1L1 subgroup changed to 1. (b) An XML file where the status from G2L2 subgroup changed to D.

The *Data Analyzer* activity examines the docking results, which are sent from FReMI by HTTP Post, based on P-SaMI data pattern. The result of these analyses is a parameter set that is stored in the wFReDoW update files (Figure 7). Thus, to keep FReMI updated with the P-SaMI results, *Data Analyzer* sends wFReDoW update files to FReMI by SFTP protocol every time P-SaMI modifies the priority and/or status of a subgroup of snapshots.

The *Database* component is based on FReDD database [26], built with PostgreSQL 4.2 (http://www.postgresql.org/docs/9.0/interactive/), and is used to provide provenance about data generated by Server Controller. The *Provenance* activity stores the Server Controller data in the Database component. Hence, the scientist is able to follow wFReDoW execution whenever he/she needs.

#### 3.1.3. FReMI: Flexible Receptor Middleware

FReMI is a middleware on the Amazon Cloud [18] that handles many tasks to execute, in parallel, the molecular docking simulations of subgroups of conformations of FFR models. It also provides the interoperability between the Server Controller layer and the virtual HPC environment built using the Amazon EC2 instances. FReMI contains five different components: *Start*, *wFReDoW Repository*, *FReMI workspace*, *FReMI execution*, and *HPC environment*. *Start* begins the execution of FReMI and *HPC Environment* denotes the virtual cluster on EC2 instances. The remaining components are described below.

The *wFReDoW Repository* contains the *Input*/*Update Files* repository. This repository stores all files sent by Server Controller layer using the SFTP network protocol. It consists of predocking files, a wFReDoW control file (Figure 6), and different wFReDoW update files (Figure 7). 

The *FReMI Workspace* component represents the directory structure used to store the huge volume of data manipulated to execute the molecular docking simulations. The input files placed in the *wFReDoW Repository* are transferred, during FReMI's execution time, to its workspace by the *Parser*/*Transfer* activity within the *FReMI Execution* set of activities. 

**Figure 8:**
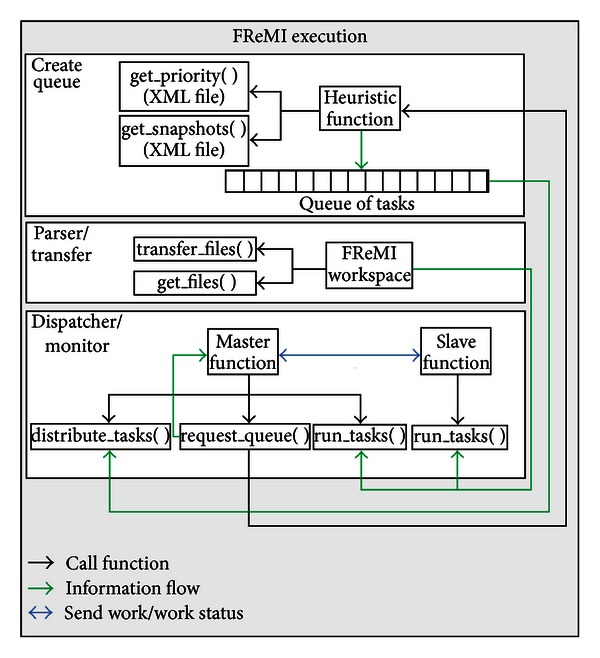
Scheme of the FReMI execution implementation. The *Create Queue*, *Parser*/*Transfer*, and *Dispatcher Monitor* components include the main functions executed by FReMI. The *Dispatcher*/*Monitor* component deals with the master-slave paradigm on the EC2 instances.

The *FReMI Execution* component—the engine of FReMI—contains every procedure invoked to run the middleware. Its source code was written in the C programming language and its libraries.  Figure 8 shows the data flow control followed by the *FReMI Execution* component. Basically, the *FReMI Execution* identifies the active snapshots (status A), inserts them in queues of balanced tasks that are created based on subgroup priorities emerging from the P-SaMI data pattern, and submits these queues into the HPC environment. These actions are performed through three activities: *Create Queue*, *Parser*/*Transfer*, and *Dispatcher*/*Monitor*.

The *Create Queue* activity produces a number of queues of balanced tasks during FReMI run time based on the information from wFReDoW control file (Figure 6). According to the priorities, this activity uses a heuristic function to determine how many processors from HPC environment will be allocated for each subgroup of snapshots. Furthermore, it uses the status to identify whether a snapshot should be processed or not. For this purpose, the *Create Queue* activity starts calculating the maximum number of snapshots that each queue can support. Thus, the amount of nodes or machines allocated (*N*) and the amount of parallel tasks (*T*) executed per node are used to obtain the queue length (*Q*), with the following equation:

(1) $$\begin{array}{c} Q=N\times T. \end{array}$$

Afterward, the amount of snapshots per subgroup is calculated in order to achieve the balanced distribution of tasks in every queue created. A balanced queue contains one or more snapshots of an active group. From the subgroup priorities, it is possible to determine the percentage of snapshots to be included in the queues. Thus, subgroups with higher priority are queued before those with lower priority. Equation (2) is used to calculate the amount of snapshots for a balanced queue:

(2) $$\begin{array}{c} S_{g}=Q\times \left(\frac{P_{g}}{\sum \left(P_{g}\right)}\right). \end{array}$$

*S*_*g*_ is the amount of snapshots of the subgroup *g* that are placed in the queue. *Q* is the queue length from (1). *P*_*g*_ is the priority of the subgroup *g*, and ∑(*P*_*g*_) is the sum of the priorities of all subgroups. From (2) one queue of balanced tasks (*B*_*q*_) is created with the following equation:

(3) $$\begin{array}{c} B_{q}=\sum \left(S_{g}\right). \end{array}$$

The *Parser*/*Transfer* activity handles and organizes the files sent by the Server Controller layer to its workspace on FReMI. It has three functions: to transfer all files received from Server Controller to the FReMI workspace by means of the *transfer file* function (see Figure 8); to perform a parse on predocking files in order to recognize the FReMI's files directory structure; and to update the parameters of the subgroups of snapshots, when necessary, using the *get files* function. The purpose of this last activity is to maintain FReMI updated with the Server Controller layer.

The functions from the *Dispatcher*/*Monitor* activity, as shown in Figure 8, are invoked to distribute tasks among the processors/cores from the virtual computer cluster on EC2 Amazon [18] based on the master-slave paradigm [23]. *Slave Function* only runs the tasks while *Master Function*, aside from running tasks, also performs two other functions: *distribute tasks*, which is activated when a node/machine asks for more work; and *request queue*, which is activated when the queue of tasks is empty. Furthermore, to take advantage of the multiprocessing of each virtual machine, we use the hybrid parallel programming model [27]. This model sends bags of tasks among the nodes by means of MPI and it shares out the tasks inside every node by OpenMP parallelization.

### 3.2. FReMI-Only Execution on Amazon EC2 MPI Cluster

**Figure 9:**
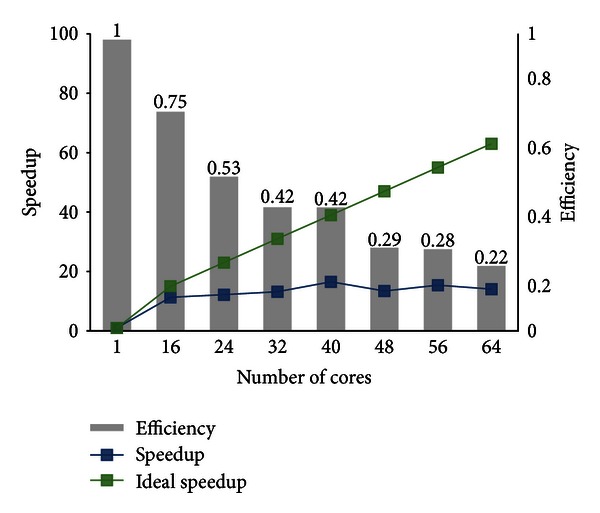
FReMI-only execution performance on Amazon EC2 determined for 3,100 docking tasks running on a scale of 1 to 8 EC2 instances.

The purpose of executing this set of experiments is to obtain the best MPI/OpenMP performance in the HPC environment on Cloud, which reduces the total elapsed time in the molecular dockings experiments, in order to become the reference to the wFReDoW experiments. For this reason, we have processed the TCL ligand (TCL400 from PDB ID: 1P45A) with two rotatable bonds against all 3,100 snapshots that make up the FFR model using FReMI-only execution. The HPC environment was executed on a scale of 1 to 8 EC2 instances. The number of tasks executed per instance was 32 (from (1): *T* = 32), and the size of the queues of balanced tasks ranged according to the number of instances used. The performance of each FReMI-only experiment versus the number of cores used is shown in Figure 9.

The performance gain obtained using the virtual MPI/OpenMP cluster on Amazon EC2 is substantial when compared to the serial version. We observed that the serial version, which was performed using only one core from an EC2 instance, took around 4 days to execute all 3,100 snapshots from the FFR model, and its parallel execution decreased this time by over 92% for the scales of cores examined. Even though the overall time of the parallel executions was reduced considerably, we also evaluated the speedup and efficiency in the virtual HPC environment to take further advantage of every core scaled during the wFReDoW execution. 

The FReMI-only execution is unable to take advantage of more than 48 cores because its efficiency ranges only from 22% to 29% (see Figure 9). Conversely, the cores were well used during the execution when we used less than 40. As can be seen, the best FReMI-only execution efficiency (i.e., 42%) was achieved using 32 and 40 cores from virtual HPC environment. However, the overall execution time spent between them was 7 hours and 28 minutes for 32 cores against 5 hours and 47 minutes for 40 cores. As a consequence of these assessments, the best FReMI-only configuration found in this set of experiments was 5 c1.xlarge EC2 Amazon instances with 8 cores each. It is worth mentioning that this configuration is able to reduce the total docking experiment time (i.e., 5 hours and 47 minutes) about 94% from its reference serial execution time, which took 90 hours and 47 minutes.

### 3.3. wFReDoW Execution on Amazon EC2 MPI Cluster

The main goal of this set of experiments is to show the performance gains in the molecular docking simulations of an FFR model and the new flexible models produced using wFReDoW. The wFReDoW experiments were conducted using 3,100 snapshots from an FFR InhA model, which are clustered by similarity functions [6], and TCL ligand (TCL400 from PDB ID: 1P45A) with two rotatable bonds. We used only an FFR model and a single ligand to evaluate wFReDoW because our goal was to analyze the performance gain in the docking experiments of FFR models by investigating the best way to coordinate, in one unique environment, all the computational techniques, such as data mining [6], data patterns for scientific workflow [10], cloud computing [18], parallel program, web server and the FReMI middleware. This variety of technological approaches contains their particular features and limits that should be dealt with in order to obtain an efficient wFReDoW implementation, avoiding fault communications, overhead, and idleness issues. Thus, from the best results, we expect that future wFReDoW executions may allow practical use of totally fully flexible receptor models playing in virtual screening of thousands or millions of compounds, which are in virtual chemical structures libraries [3], such as ZINC database [19].

According to the P-SaMI data pattern, the analyses start after a percentage of snapshots has been docked. In these experiments we seek to know how many snapshots are discarded and what the quality is of the RFFR models which are produced for each clustering when the P-SaMI data pattern starts to evaluate after 30%, 40%, 50%, 70%, and 100% of the docked snapshots. When 100% of snapshots are docked P-SaMI does not analyze the docking results. Thus, we perform fifty different kinds of docking experiments—one P-SaMI configuration for each clustering of snapshots. In this sense, Server Controller prepared three different wFReDoW control files—one for each clustering of snapshots generated by [6]—and four different P-SaMI configurations followed the above mentioned percentage.

**Figure 10:**
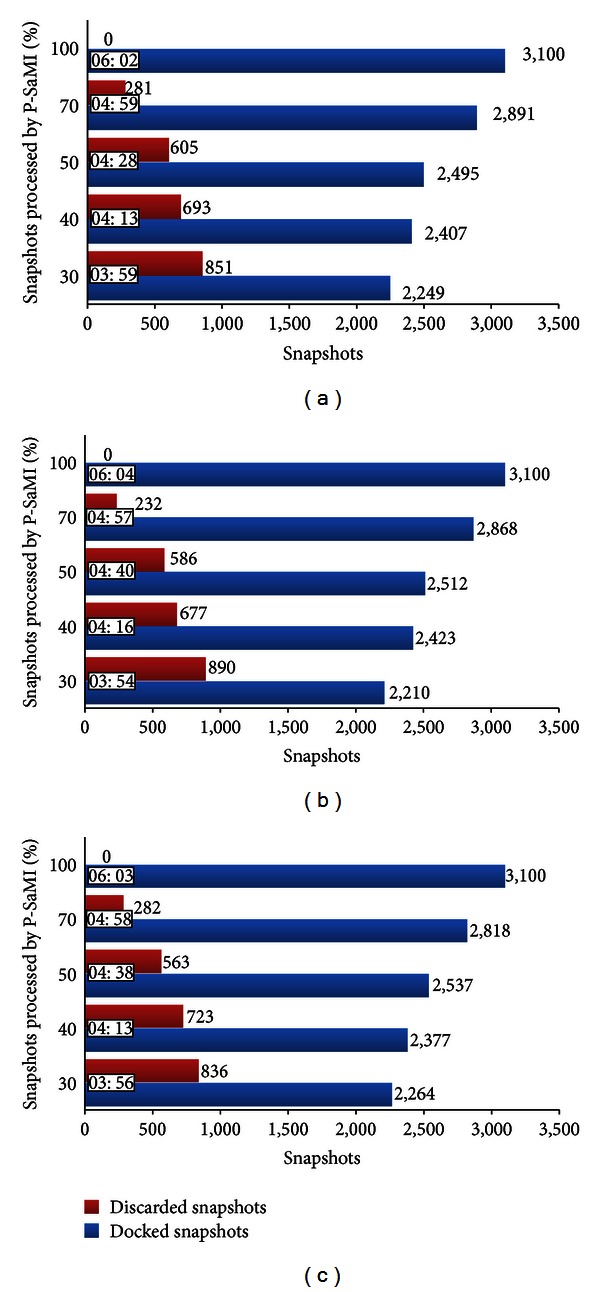
Results of the wFReDoW experiments using a different P-SaMI configuration for each clustering of snapshots. Docked snapshots (blue bar) are examples of RFFR models. (a) wFReDoW results for clustering 01. (b) wFReDoW results for clustering 02. (c) wFReDoW results for clustering 03.

Figure 10 summarizes the total execution time and the number of snapshots docked and discarded for each wFReDoW experiment. In this Figure, each graph represents the wFReDoW results obtained by running a P-SaMI configuration for each clustering of snapshots, which are represented by 01, 02, and 03 clustering. Every clustering contains 3,100 snapshots from the FFR model, which are grouped from 4 to 6 clusters depending on the similarity function used by [6]. The total time execution for each experiment (one clustering for one P-SaMI configuration) is calculated from the moment the preparation of the wFReDoW control file (in the Server Controller) begins, until the last docking result comes in the Server Controller.

## 4. Discussion 

In this paper we presented the roles of wFReDoW—a cloud-based web environment to faster execute molecular docking simulations of FFR models—and, through its execution, we showed the RFFR models produced. As can be observed in Figure 10, wFReDoW, as well as creating new RFFR models, also speeds up the docking experiments for all cases due to the reduction of docking experiments provided by the P-SaMI data pattern and the simultaneous docking execution performed by the virtual HPC environment. Although we use a small FFR model and only a single ligand, it is clear to see that wFReDoW is a promising tool to start performing molecular docking simulations for new FFR models even using large libraries of chemical structures for the practice of virtual screening.

### 4.1. wFReDoW Performance

According to [10], the earlier the analysis starts (in this case 30%), the larger the quantity of unpromising snapshots that can be recognized and discarded is. Figure 10 evidences this statement. The wFReDoW results show that when P-SaMI data pattern starts the analyses of the FFR model with 30% of docked snapshots, the number of unpromising snapshots discarded is higher. Additionally, as this percentage increases, the number of unpromising docked snapshots increases as well. Consequently, if the number of docked snapshots decreases, the overall execution time also decreases. Thus, considering the best run time of wFReDoW, that is, 3 hours and 54 minutes (Figure 10), the gain achieved by the use of P-SaMI showed a fall of 30% from the FReMI-only overall execution (5 hours and 47 minutes). 

Another consideration for wFReDoW performance is that FReMI middleware also runs in local cluster infrastructure. However, the efficiency is not the same. We also executed FReMI only using a sample of snapshots from the FFR InhA model on the Atlantica cluster with the intention to compare the performance gains obtained between the virtual and the local cluster infrastructures (Atlantica cluster consists of 10 nodes connected by a fast network system. Each node contains two CPUs Intel Xeon Quad-Core E5520 2.27 GHZ with Hyper-Threading, and 16 GB of RAM, aggregating 16 cores per node. The cluster is connected by a two-gigabit Ethernet network, one for communication between nodes and another for management. Atlantica cluster supplies high performance computational resources for the academic community.) We made several investigations for different nodes and core scales, even for different numbers of tasks executed per node. At the end we found that, in most cases, Amazon EC2 outperforms the Atlantica cluster. For instance, using the same number of cores from Amazon EC2, that is, 5 nodes with 8 cores each, for a sample of 126 snapshots from the FFR model and 16 tasks executed per instance (from (1): *T* = 16), the total execution time was 14.94 minutes for the Atlantica cluster and 8.78 minutes for Amazon EC2. Possibly, this performance difference is because we used the Atlantica cluster in a nonexclusive mode, sharing the cluster's facilities. From this evidence and our previous studies, we concluded that the EC2 configuration bestows itself as a very attractive HPC solution to execute molecular docking simulations of a larger set of snapshots and for different ligands.

### 4.2. The Quality of the RFFR Models Produced

We showed that the approach used in this study enhances the performance of the molecular docking simulations of FFR models in most cases. However, to make sure that the P-SaMI data pattern selected the best snapshots from the cluster of snapshots used, we verified the quality of the RFFR models built by wFReDoW. Regarding this, we took only the first run of the 25 runs performed by AutoDock 4.2, which contains the best FEB of each docking, to evaluate the produced models. The best docking result of each snapshot was organized according to the percentage of snapshots with the best FEB values in an ascending order (set of best FEB). Then, we investigated if the selected snapshots belonged to the percentage of this set. As a result we obtained the data described in Table 1 with the number of docked snapshots for each set of best FEB and its respective accuracy. 

Based on the data illustrated in Table 1 we can observe that wFReDoW worked well for all P-SaMI analyses. This is evidenced from the computed accuracy in the produced RFFR models, which contain more than 94% of its snapshots within the set of best FEB values. In the clustering 02, for instance, when P-SaMI started the analysis in 70%, wFReDoW worked best, selecting 308 of the 310 best ones, 612 of the 620 best ones, and 913 of the 930 best ones. Whilst, when P-SaMI started the analysis in 30% in the same clustering, wFReDoW selected 302 of the 10% best ones, 593 of the 20% best ones, and 871 of the 30% best ones. Even though wFReDoW selected fewer snapshots in the latter P-SaMI configuration, it represents 97.42%, 95.65% and 93.66% of the 10%, 20%, and 30% best FEB, respectively. The difference between the best and worst wFReDoW selections is slight. However, the difference between them of 1 hour in the total wFReDoW execution time (3 hours and 54 minutes for P-SaMI analysis from 30% against 4 hours and 57 minutes for P-SaMI analysis from 70%) could be a good motivation to start the P-SaMI analyses when only 30% of the snapshots have been docked. Consequently, it also is a promising opportunity for reducing the overall execution time and preserving the quality of the models produced.

It is worth mentioning that wFReDoW is only capable of building an RFFR model, without losing the quality of its original model, if the clustering methods used as input data contain high affinity among the produced clusters of snapshots from [6]. This means that wFReDoW, with its features, is always able to improve the performance. However, for improving the quality of the RFFR models produced, the used clustering also needs to be of a high quality.

### 4.3. Amazon Cloud

The most significant advantage of shared resources is the guaranteed access time of the resources wherever you are and whenever you need. There is no competition or restrictions for access to the machines. However, it is necessary to pay for as many computing nodes as needed, which are charged at an hourly rate. The rate is calculated for what resources are being used and when; for example, if you do not need computing time, you do not need to pay.

## 5. Conclusions 

The main contribution of our article is wFReDoW, a cloud-based web environment to faster handle molecular docking simulations of FFR models using more than one computational approach cooperatively. wFReDoW includes the P-SaMI data pattern to select promising snapshots and the FReMI middleware that uses an HPC environment on the Amazon EC2 instances to reduce the total elapsed time of docking experiments. The results showed that the best FReMI-only performance decreased the overall execution time by about 94% with its respective serial execution. Furthermore, wFReDoW reduced the total execution time a further 10–30% from FReMI-only best execution without affecting the quality of the produced RFFR models.

There are several possible ways to further improve the efficiency of wFReDoW. One of the biggest limitations for wFReDoW's performance is that the Server Controller layer runs in a web server located outside of Amazon EC2. Even though we posted all docking input files inside wFReDoW repository (inside FReMI layer) in advance, there are still a large number of files that are transferred during the wFReDoW execution. In this experiment, the time taken to transfer these files was irrelevant since our FFR model holds only 3,100 snapshots. However, when using FFR models with hundreds to thousands of snapshots, the time will be increased significantly. A way to enhance the overall performance is by the use of an EC2 instance to host the Server Controller layer. This would greatly reduce the time taken to transfer the files from Server Controller to FReMI. Furthermore, the Server Controller layer could also send only the docking input files from promising snapshots during the wFReDoW execution, contributing to the reduction in the amount of files transferred and in the overall elapsed time.

wFReDoW was tested with a single ligand and an FFR model containing only 3,100 conformations of InhA generated by an MD simulation. MD simulations are now running on tens to hundreds of nanoseconds for the same model. This could produce FFR models with more than 200,000 snapshots! wFReDoW should be tested with such models. Additionally, it would be interesting to make use of other ligands by means of investigation of public databases of small molecules, such as ZINC [19]. 

## Figures and Tables

**Table 1:** Analysis of the wFReDoW results obtained by running a P-SaMI configuration for each clustering of snapshots. Column 1 identifies the three different types of clustering. Column 2 specifies the percentage of docked snapshots after which P-SaMI analysis of the model quality starts. Columns 3, 5, and 7 display the total number of selected snapshots that are in the best 10%, best 20%, and best 30%, respectively. Columns 4, 6, and 8 present the accuracy percentage for the best 10%, 20%, and 30%, respectively.

Clustering	P-SaMI	Best 10%	Accuracy %	Best 20%	Accuracy %	Best 30%	Accuracy %
01	30%	305	98.39	598	96.45	879	94.52
01	40%	305	98.39	600	96.77	887	95.38
01	50%	306	98.71	603	97.25	894	96.13
01	70%	308	99.35	608	98.06	910	97.85
02	30%	302	97.42	593	95.65	871	93.66
02	40%	302	97.42	599	96.61	888	95.48
02	50%	303	97.74	599	96.61	891	95.81
02	70%	308	99.35	612	98.71	913	98.17
03	30%	300	96.77	596	96.13	885	95.16
03	40%	301	97.10	599	96.61	891	95.81
03	50%	303	97.74	604	97.42	898	96.56
03	70%	303	97.74	610	98.39	909	97.74
—	100%	310	100.00	620	100.00	930	100.00
